# Suppression of AMD-Like Pathology by Mitochondria-Targeted Antioxidant SkQ1 Is Associated with a Decrease in the Accumulation of Amyloid β and in mTOR Activity

**DOI:** 10.3390/antiox8060177

**Published:** 2019-06-14

**Authors:** Natalia A. Muraleva, Oyuna S. Kozhevnikova, Anzhela Z. Fursova, Nataliya G. Kolosova

**Affiliations:** 1 Institute of Cytology and Genetics SB RAS, Pr. Lavrentyeva 10, Novosibirsk 630090, Russia; Myraleva@bionet.nsc.ru (N.A.M.); oidopova@bionet.nsc.ru (O.S.K.); anzhellafursova@yandex.ru (A.Z.F.); 2 N. N. Vorozhtsov Novosibirsk Institute of Organic Chemistry SB RAS, 9 Lavrentieva Avenue, Novosibirsk 630090, Russia

**Keywords:** age-related macular degeneration, mitochondria-targeted antioxidant SkQ1, amyloid beta, mTOR, OXYS rats

## Abstract

Age-related macular degeneration (AMD) is a major cause of irreversible visual impairment and blindness in developed countries, and the molecular pathogenesis of AMD is poorly understood. Recent studies strongly indicate that amyloid β (Aβ) accumulation —found in the brain and a defining feature of Alzheimer’s disease—also forms in the retina in both Alzheimer’s disease and AMD. The reason why highly neurotoxic proteins of consistently aggregate in the aging retina, and to what extent they contribute to AMD, remains to be fully addressed. Nonetheless, the hypothesis that Aβ is a therapeutic target in AMD is debated. Here, we showed that long-term treatment with SkQ1 (250 nmol/[kg body weight] daily from the age of 1.5 to 22 months) suppressed the development of AMD-like pathology in senescence-accelerated OXYS rats by reducing the level of Aβ and suppressing the activity of mTOR in the retina. Inhibition of mTOR signaling activity, which plays key roles in aging and age-related diseases, can be considered a new mechanism of the prophylactic effect of SkQ1. It seems probable that dietary supplementation with mitochondria-targeted antioxidant SkQ1 can be a good prevention strategy to maintain eye health and possibly a treatment of AMD.

## 1. Introduction

Age-related macular degeneration (AMD) is the leading cause of irreversible visual impairment and blindness in industrialized countries. The prevalence of AMD is increasing dramatically as the proportion of the elderly in the population continues to rise. AMD is classified into dry AMD and wet AMD depending on the presence of choroidal neovascularization. Although the introduction of anti-angiogenesis therapy has helped to prevent blindness and restore vision in wet AMD, there remains no effective treatment for the majority of patients with dry or nonexudative AMD [1]. The pathogenesis of this complex degenerative disease includes both genetic and epigenetic risk factors but is not completely clear. The main pathological changes that drive AMD are inflammation, endoplasmic reticulum stress, and oxidative stress [2]. Reactive oxygen species (ROS) are produced mainly in mitochondria, whose dysfunction, in combination with defective proteostasis, is a hallmark of age-related degenerative diseases including AMD and Alzheimer’s disease (AD) [3]. The research to date suggests that antioxidant supplements (at least those studied) do not prevent and certainly do not suppress the progression of AMD [4,5]. Perhaps this phenomenon is due to low bioavailability (especially of conventional antioxidants) in the retina, which is isolated from the bloodstream. Besides, mitochondria have low permeability to conventional antioxidants, and the latter have low effectiveness against ROS formation in these organelles [6]. These shortcomings have prompted the creation of a new class of mitochondria-targeted antioxidants in the last decade, which are considered a promising intervention in age-related ocular diseases [7]. One of these compounds is SkQ1 (10-(6′-plastoquinonyl) decyltriphenyl-phosphonium), a plastoquinol derivative modified by a lipophilic cation, which enables penetration of this drug through the mitochondrial inner membrane and its accumulation in the mitochondrial matrix. The ability of SkQ1 to slow down aging and to retard, arrest, and in some cases even reverse the development of many age-related health problems has been proved on different animal models [8,9]. In the form of eye drops, SkQ1 has shown efficacy in the prophylaxis and/or treatment of cataract [10,11], glaucoma, and uveitis, as well as corneal lesions and dry eye syndrome of different etiologies [12,13,14]. Our data have revealed that dietary supplementation with SkQ1, as well as SkQ1 eye drops, can prevent the development and cause regression of pre-existing signs of the AMD-like retinopathy in senescence-accelerated OXYS rats [8,12,15]. Retinopathy that develops in OXYS rats already at a young age corresponds to the dry atrophic form of AMD in humans. The clinical signs of retinopathy in the development of OXYS rats by the age of three months against the background of a reduction in the transverse area of the retinal pigment epithelium (RPE), retinal thinning, and impairment of choroidal microcirculation [16]. Progression of these abnormalities in OXYS rats is manifested by a significant reduction in the thickness of the photoreceptor cell layer [17]. Significant pathological changes in the RPE are accompanied as excessive accumulation of lipofuscin [18] and amyloid beta (Aβ) [19] in the RPE regions, disturbances in the morphology of the RPE sheet (including an increase in the proportion of multinucleated cells), a distortion of cell shape, and reactive gliosis and hypertrophy [20]. We reported that the beneficial effects of SkQ1 on AMD-like retinopathy are related to the prevention of ultrastructural alterations in the RPE, attenuation of neurodegenerative changes in photoreceptors, facilitation of circulation in choroid blood vessels, and normalization of VEGF and PEDF expression in the retina of OXYS rats, and as a consequence, an improving b-wave response [12,16,18,21]. Nevertheless, the mechanisms of SkQ1′s action are not completely understood, and more research efforts are needed to identify the signaling pathways influenced by this compound. Previously, we found that the autophagic pathway participates in the progression of AMD-like retinopathy in OXYS rats [22]. One of the consequences of autophagy aberrations may be significant accumulation of Aβ_1–42_ in aged OXYS rats’ retina [23]. This protein fragment is a member of a highly toxic and aggregation-prone family of peptides that is upregulated in the ageing retina and is associated with AMD [24,25]. Accumulation of Aβ is also observed in the brain of OXYS rats and is regarded by us as one of the manifestations of the AD-like pathology that can be partially retarded by SkQ1 [9,26,27]. A crucial role in the regulation of autophagy is played by mammalian/mechanistic target of rapamycin (mTOR), which integrates the intracellular signals that control cell growth, nutrient metabolism, and protein synthesis and regulates mitochondrial biogenesis to coordinate energy homeostasis with cell growth [28,29,30]. Compelling evidence indicates that the mTOR signaling pathway is deregulated in aging and age-related neurodegenerative diseases [31], and activation of mTOR signaling is a contributor to AD progression [32]. It is believed that alterations of the mTOR pathway contribute to the pathogenesis of AMD, but there are no data on the alterations of its activity in the retina, and this information is limited to a few studies largely focused on retinal cells in the RPE [33]. Previously, we showed that treatment with mTOR inhibitor rapamycin in a dose-dependent manner decreases the incidence and severity of retinopathy in OXYS rats in the period of active manifestation of AMD signs (age from 1.5 to 3.5 months) and demonstrated the potential of the mTOR activity suppression strategy for the treatment and prevention of AMD [34]. Here, we report that long-term treatment with SkQ1 (250 nmol/[kg body weight] daily from the age of 1.5 to 22 months) suppressed the development of the AMD-like pathology in OXYS rats by reducing the level of Aβ and suppressing the activity of mTOR in the retina.

## 2. Materials and Methods

### 2.1. Ethics Statement

All animal procedures were in compliance with the Association for Research in Vision and Ophthalmology statement for the Use of Animals in Ophthalmic and Vision Research and the European Communities Council Directive 86/609/EES. All manipulations of the animals were approved by Scientific Council 9 of the Institute of Cytology and Genetics, the Siberian Branch of the Russian Academy of Sciences, according to The Guidelines for Manipulations with Experimental Animals (the decree of the Presidium of the Russian Academy of Sciences No. 12000-496 of April 2, 1980).

### 2.2. Animals and Diet

Male senescence-accelerated OXYS rats (n = 30) and age-matched male Wistar rats (control, n = 30) were obtained from the Center for Genetic Resources of Laboratory Animals at the Institute of Cytology and Genetics, the Siberian Branch of the Russian Academy of Sciences (RFMEFI61914 × 0005 and RFMEFI61914X0010). At the age of four weeks, the pups were weaned, housed in groups of five animals per cage (57 × 36 × 20 cm), and kept under standard laboratory conditions (22 °C ± 2 °C, 60% relative humidity, and 12 h light/12 h dark cycle; lights on at 9 a.m.). The animals were provided with standard rodent feed (granulated food for laboratory animals; Chara, Assortiment-Agro, Russia) and water *ad libitum*.

To study the influence of dietary SkQ1 supplementation on retinopathy development, 1.5-month-old OXYS and Wistar rats were randomly assigned to one of two groups (n = 15): A control diet or control diet supplemented with SkQ1 (Institute of Mitoengineering of Moscow State University (Moscow, Russia) 250 nmol/(kg of body weight) per day from the age of 1.5 to 22 months. OXYS rats are characterized by lower body weight in comparison with Wistar rats. Accordingly, before treatment at the age of 1.5 months, the body weight was dependent only on the genotype (F_1,58_ = 28.7, *p* < 0.00002) and was lower in OXYS rats (153 ± 4 vs. 189 ± 7 g). At the end of the 21-month treatment with SkQ1, the body weight remained lower in OXYS rats (424 ± 10 vs. 577 ± 14 g; F_1,59_ = 176, *p* < 0.000) and was not affected by the antioxidant (F_1,59_ = 0.59, *p* = 0.445).

### 2.3. Ophthalmoscopic Examination

**Figure 1:**
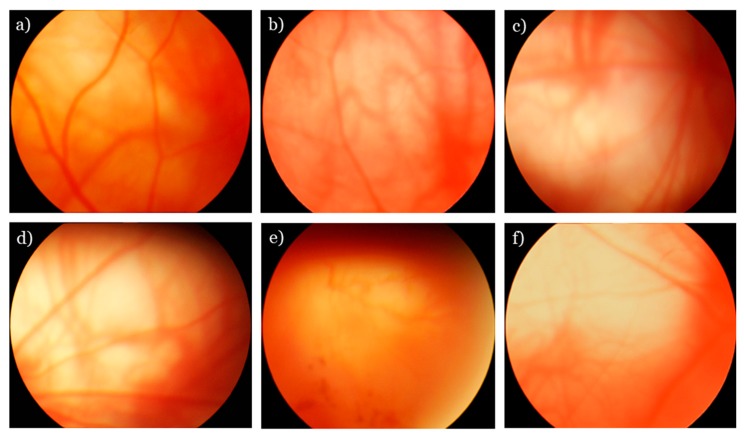
Fundus photographs of rat retinas. (**a**) A normal fundus from Wistar rat. The proportion of blood vessels is normal. The retina between vascular arcades is not damaged (or changed). (**b**–**f**) The fundus images from OXYS retinas: (**b**) A combination of multiple small drusen and a few medium-size drusen; this condition corresponds to 1 AU; (**c**) numerous intermediate reticular drusen without pigmentary irregularities; this condition corresponds to 2 AU; (**d**) obliteration of choroidal vessels, destruction and sclerosis of retinal vessels, and a sharply demarcated (usually round or oval) area of atrophy of the retinal pigment epithelium (RPE); this condition corresponds to 2 AU; (**e**) intraretinal hemorrhages with edema and serous detachment of the neurosensory retina and RPE, with neovascularization; this condition corresponds to 2 AU; (**f**) geographic atrophy of the RPE (3 AU).

All the rats were examined by an ophthalmologist four times: Before supplementation at the age of 1.5 months, and during the treatment at ages 3, 12, and 22 months. All the rats underwent funduscopy with a Heine BETA 200 TL Direct Ophthalmoscope (Heine, Herrsching, Germany) after dilatation with 1% tropicamide. An assessment of stages of retinopathy was performed according to the Age-Related Eye Disease Study grade protocol. A Kowa Genesis-D fundus camera (Japan) was used as a handheld digital camera to take fundus photographs of the retina. The degree of retinopathy was estimated as follows: 0 arbitrary units (AU) corresponded to healthy retina; 1 AU, appearance of drusen, and other pathological changes in the RPE and partial atrophy of the choroid capillary layer; 2 AU, exudative detachment of the RPE and of the retinal neuroepithelium, with further choroid capillary layer atrophy; and 3 AU, neovascularization and exudative hemorrhagic detachment of the RPE and neuroepithelium scarring. The examples of alterations of fundus oculi in OXYS rats are shown in Figure 1. Five days after the last eye examination, the rats were euthanized by CO_2_ asphyxiation and decapitated. Three eyes from each group were excised and fixed for histopathological examination. The retina of eyes was separated from the other tissues, placed in microcentrifuge tubes for protein isolation, and frozen in liquid nitrogen. All the specimens were stored at −70 °C before analysis. 

### 2.4. Histopathological Investigation

For analysis of the main indicators of destruction in the retina of OXYS rats and Wistar rats and for evaluation of effects of the SkQ1, the fundus of the eyes was fixed in Carnoy’s fluid (absolute alcohol, chloroform, and glacial acetic acid in the ratio 6:3:1) for 4 h, and then washed for several hours in absolute alcohol until disappearance of the acetic-acid smell, was compacted, and embedded in paraffin by the standard method. Using a rotary microtome, we prepared vertical sections (4–5 µm thick) of the eye fundus and stained them with hematoxylin and eosin. Five slices of each retina were used for histopathological examination. The slides were masked. Examination and imaging of the slices were conducted under the microscope Carl Zeiss Axiostar plus. In the resulting images, by means of the Carl Zeiss AxioVision 8.0 software at magnification 10 × 100, we calculated the average area of retinal pigment epithelium (RPE) cell cytoplasm in the retinal cross-section. Using an Avtandilov grid, we determined the number of layers nuclei in the outer nuclear layer in the ocular frame within an area of 1 mm^2^ from five slices of each retina at magnification 10 × 100. We counted neurons with nuclear pyknosis per 200 corresponding cells in each retina. 

### 2.5. Western Blotting and an ELISA

Frozen tissues of the retina of OXYS rats and Wistar rats and for evaluation of effects of the SkQ1 (n = 5) were homogenized in lysis (RIPA, radioimmunoprecipitation assay) buffer (50 mmol/L Tris-HCl, pH 7.4; 150 mmol/L NaCl; 1% of Triton X-100; 1% of sodium deoxycholate; 0.1% of SDS; and 1 mmol/L EDTA) supplemented with a protease inhibitor cocktail (P8340; Sigma-Aldrich, St. Louis, MO, USA). After incubation for 20 min on ice, the samples were centrifuged at 9660× *g* at 4 °C for 30 min, and the supernatants were transferred to new tubes. Total protein was quantified with the Bradford assay kit (Bio-Rad Laboratories, Hercules, CA, USA). Samples were resolved by SDS-PAGE on 12% gels in Tris-glycine buffer (25 mmol/L Tris base, 190 mmol/L glycine, and 0.1% of SDS) and transferred to nitrocellulose membranes. The membranes were probed with specific antibodies against S6 ribosomal protein and phospho-S6 ribosomal protein (p-S6) (1:1000; Cell Signaling Technology, Danvers, MA, USA) at 4 °C overnight with an anti-actin antibody (cat. # ab1801; 1:1000; Abcam, Cambridge, MA, USA). Signals were scanned and the intensity of the bands was measured in ImageJ, version 1.41 (NIH, Bethesda, MD, USA). 

The Human/rat β Amyloid (42) ELISA Kit (Wako, Japan) was employed to measure Aβ levels in the retina of all the rat groups according to the manufacturer’s instructions.

### 2.6. Immunohistochemistry

The right eyes of OXYS rats from control and SkQ1-treated groups (n = 4) were removed and fixed in fresh 4% paraformaldehyde in PBS for 2 h, washed three times in PBS, and then cryoprotected in graded sucrose solutions (10%, 20%, and 30%). Posterior eyecups were embedded in Killik (Bio-Optica), frozen, and stored at −80 °C. Tissue slices (10 µm thick) were prepared on a Microm HM-505N cryostat at −20 °C, transferred onto Polysine^®^ glass slides (Menzel-Glaser), and stored at −20°C. After several washes in PBS with 0.1% Triton X-100 (PBST), the slices were incubated for 1 h in 5% BSA in PBST, followed by overnight incubation at 4 °C with the rabbit antibodies to Aβ_1–42_ (1:50 dilution, Abcam) and to p-S6 (1:150 dilution, Cell Signaling Technology). After three washes in PBST, the tissue slices were incubated with the secondary antibody conjugated with Alexa Fluor^®^ 488 (Abcam) at a dilution of 1:300 for 1 h and next washed with PBST. The slices were coverslipped with the Fluoroshield mounting medium supplemented with DAPI (Abcam) and examined under an Axioplan 2 microscope (Zeiss). For acquisition of each image, all imaging parameters were the same.

### 2.7. Statistical Analysis

The data were subjected to ANOVA (Statistica 8.0 software). The Newman–Keuls test was applied to significant main effects and interactions in order to assess the differences between some sets of means. The data were presented as mean ± SEM. The differences were considered statistically significant at *p* < 0.05.

## 3. Results

### 3.1. SkQ1 Inhibits Retinopathy Development in OXYS Rats

**Figure 2:**
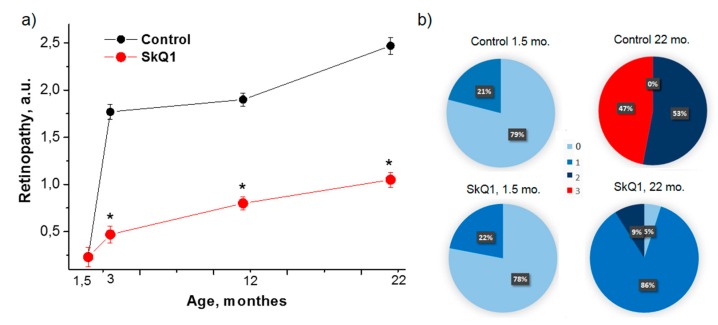
SkQ1 inhibits retinopathy development in OXYS rats. The age-dependent changes in severity of retinopathy in control and SkQ1 treated OXYS rats; the treatment (250 nmol/[kg body weight] daily) was started when the rats were 1.5 months old (**a**). Each group corresponds to 30 eyes of 15 animals. The data are presented as mean ± SE of a.u. corresponding to the stages of retinopathy. Stages of retinopathy in control and SkQ1-treated OXYS rats at the age of 1.5 and 22 months (**b**). Data are presented as percentage of eyes with stage 0, 1, 2, or 3 of retinopathy.

Preliminary examination showed that there was no difference between 1.5-month-old OXYS rats assigned to experimental and control groups: 22% and 21% of the rats had signs of stage 1 retinopathy, respectively. By the age of 22 months, 53% and 47% of control OXYS rats developed stage 2 and 3 retinopathy, respectively (Figure 2). SkQ1 decreased the incidence and severity of retinopathy. Thus, only 9% of the rats treated with SkQ1 developed stage 2 retinopathy, 86% of the rats developed stage 1 retinopathy, and 5% of the rats remained disease-free (Figure 2). In Wistar rats, which do not naturally develop retinopathy, repeated inspections did not reveal pathological alterations in the retina of SkQ1-treated rats. 

### 3.2. SkQ1 Prevents Neurodegeneration as Assessed by Histological Examination

**Figure 3:**
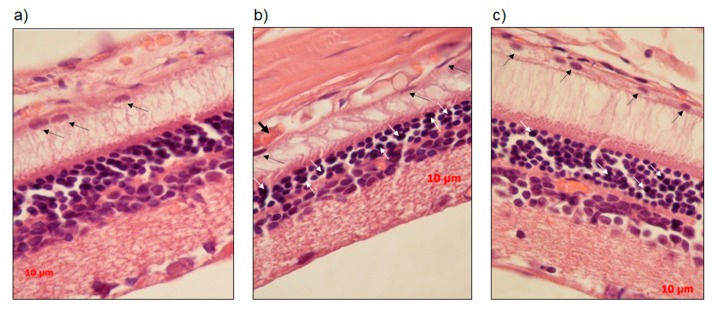
Representative images of hematoxylin and eosin staining from central retina of Wistar rats (**a**), OXYS rats (**b**) and OXYS treated with SkQ1 (**c**). The neuron with pyknosis (white arrow); the nucleus of the RPE cell (black dotted arrows); stasis and sludge of the blood cells in capillaries of the choroid (black arrow). The SkQ1 significantly reduced the percentage of neurons with pyknosis, ameliorated atrophic changes in RPE and choroid (**c**). The scale bar is 10 µm.

We next compared the histological features of the retina in OXYS and Wistar rats. Unlike the choroid of Wistar rats (Figure 3a), the choroid of OXYS rats exhibited disturbances of blood flow: Aggregation of blood cells, thrombosis, and stasis of small vessels (Figure 3b). In OXYS rats, there were aberrations of RPE cells–flattening, with a variable size and shape of their nuclei, typical to AMD. We detected vacuolization of the cytoplasm neurons and nuclear pyknosis of neuroretina cells. SkQ1 decreased significantly of the proportion of neurons with pyknosis (from 10.6 ± 2.3 to 5.6 ± 1.4, %, *p* < 0.05), significantly increased in the transverse area of RPE (from 75.3 ± 4.2 to 93.1 ± 6.10, µm^2^, *p* < 0.05) and increased the number of photoreceptor rows (4.2 ± 0.8 to 5.4 ± 0.4, ns *p* > 0.05) (Figure 3c). 

### 3.3. SkQ1 Inhibits mTOR

**Figure 4:**
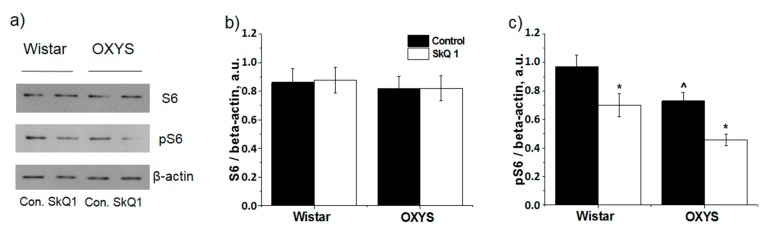
Effects of treatment with SkQ1 (250 nmol/kg per day from 1.5 to 22 months of age) on the protein levels of ribosomal protein S6 and p-S6 in the retina of OXYS and Wistar rats. The levels of S6 and p-S6 in the retina of OXYS and Wistar rats, according to western blot analysis (**a**). The relative quantity of proteins S6 (**b**) and p-S6 (**c**) was calculated with β-actin as the control (M ± SE, n = 5). ^: Statistically significant differences between the strains of the same age; * the effect of SkQ1 (*p* < 0.05).

Next, we investigated phosphorylation of S6 (p-S6), an indicator of the mTOR pathway activity. There was no significant difference between the levels of S6 in the retina of OXYS and Wistar rats; S6 levels were not affected by the treatment with SkQ1 (Figure 4a,b). The level of p-S6 in the retina of the untreated 22-month-old OXYS rats was 24% lower than that in Wistar rats (*p* < 0.036). SkQ1 decreased phosphorylation of S6 in OXYS and Wistar rats by 39% (*p* < 0.004) and 28% (*p* < 0.023), respectively, indicating a decrease in mTOR activity caused by SkQ1 (Figure 4a,c). The level of phosphorylation stayed lower in OXYS rats than in Wistar rats.

### 3.4. SkQ1 Prevents Accumulation of Aβ

**Figure 5:**
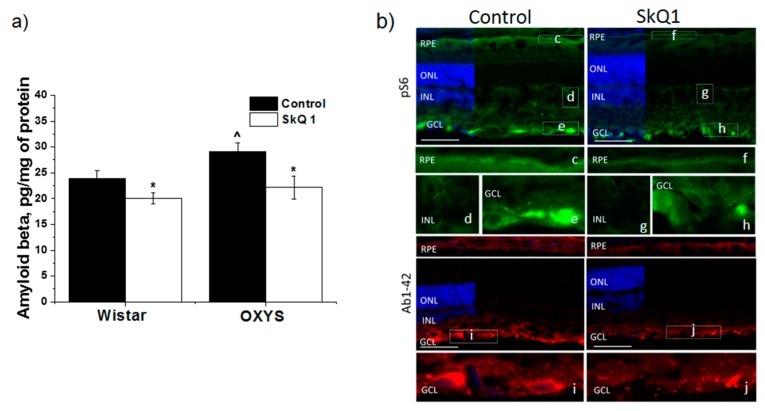
The effect SkQ1 (250 nmol/[kg of body weight] per day from the age of 1.5 to 22 months) on the Aβ and p-S6 protein in the retina of OXYS rats and Wistar. Immunoassay revealed decrease Aβ1-42 protein content in the retina of OXYS rats and Wistar rats treated with SkQ1 (**a**). * *p* < 0.05: a statistically significant effect of SkQ1; ^ *p* < 0.05: a difference between OXYS and Wistar rats (mean ± SE). Representative images of retinal cryosections immunostained for p-S6 and Aβ1–42 and enlarged images from insets in RPE, INL and GCL (**b**). Immunostaining for p-S6 (green) uncovered a decrease in the intensity of p-S6-positive cells in the retina of OXYS rats treated with SkQ1. SkQ1 treatment also reduced the Aβ1–42 (red) immunoreactivity in the retina of OXYS rats. Cell nuclei were stained with DAPI. RPE: Retinal pigment epithelium, ONL: Outer nuclear layer, INL: Inner nuclear layer, GCL: Ganglion cell layer. The scale bar is 50 μm.

The level of Aβ_1–42_ in the retina of the untreated OXYS rats was higher than that in Wistar rats (*p* < 0.029; Figure 5a). After treatment with SkQ1, in the retina of OXYS rats and Wistar rats, we observed a decrease in the level of Aβ_1–42_ compared the untreated groups (*p* < 0.020 and *p* < 0.049, respectively; Figure 5a). 

We next examined the effects of SkQ1 on retinal mTOR activity by immunohistochemical analysis (Figure 5b). Staining of p-S6, a downstream target of mTOR, was found in the regions of the ganglion cell layer (GCL), inner nuclear and inner plexiform layers, and the RPE. SkQ1 treatment reduced the p-S6 immunoreactivity in the GCL. 

Aβ staining was evident in retinal cryosections of both control and SkQ1-treated OXYS rats and was predominantly limited to the RPE and blood vessels in the choroid and in the GCL. Aβ immunoreactivity decreased after SkQ1 treatment.

## 4. Discussion

Recent studies strongly indicate that Aβ, found in the brain and a defining feature of AD, also forms in the retina in both AD and AMD [35,36]. The reason why proteins of this highly neurotoxic family consistently aggregate in the aging retina, and to what extent they contribute to AMD, remains to be fully addressed [25]. Nevertheless, the hypothesis that Aβ is a therapeutic target in AMD is debated. Previously, we reported age-dependent upregulation of Aβ in the retina and in the brain of OXYS rats, which spontaneously develop all the major signs of both AMD and AD [37]. We also found that dietary supplementation with SkQ1 suppresses the development of the key signs of accelerated senescence in 24-month-old OXYS rats, including AD-like pathology [26] and AMD-like pathology [8,12,38]. Ophthalmoscopy observations were confirmed by histological analysis of the retina, as well as by our observations that the electroretinogram disappeared in the majority of the 24-month-old OXYS rats, but was retained in OXYS rats with SkQ1 as well as in Wistar rats (SkQ1 increased the b-wave magnitude of the electroretinogram from 19 ± 13 to 42 ± 9 µV in OXYS rats and from 37 ± 9 to 55 ± 12 µv in Wistar rats) [8,12,38]. Here, we confirmed that dietary supplementation with SkQ1 starting at the age of 1.5 months suppresses the development of the AMD-like retinopathy in old OXYS rats, and for the first time, showed that its effects are associated with a decrease in the accumulation of Aβ and in mTOR activity. Treatment with SkQ1 significantly decreased the activity of mTOR signaling in the retina of both OXYS and Wistar rats according to downregulation of the most widely used biochemical marker of mTOR activity, p-S6, which is a substrate of mTORC1, one of two complexes of mTOR.

The key regulator of Aβ generation and clearance is autophagy, which is dysregulated in AMD [39,40,41,42]. Our previous results point to disturbances in the autophagy process in the retina of OXYS rats: its activation in the early stages of the disease and suppression in late stages [22]. The decrease in autophagic activity in the retina with age leads to inefficient clearance of outer segments, whereas damaged mitochondria accelerate the accumulation of the Aβ group of misfolded proteins and of lipofuscin [25]. Enhanced accumulation of lipofuscin and Aβ in the retina, accompanied by the decrease in autophagy, are characteristic of both OXYS rats [18,19] and humans with late-stage AMD [43]. We propose that such alterations may be due to activation of mTOR signaling, which is considered a contributor to the progression of AD and of other neurodegenerative diseases [31]. On the other hand, we revealed that the activity of the mTOR pathway in the retina of untreated 23-month-old OXYS rats was lower as compared to age-matched Wistar control rats (in the present study) and in the retina of 3-month-old OXYS rats (slowly but significantly: By 13%, *p* < 0.003, as we demonstrated earlier [44]). mTOR is a ubiquitously expressed kinase, but its regulation is tissue specific and cell type specific. mTOR has been repeatedly shown to participate in neuronal development and the proper functioning of mature neurons. mTOR-controlled signaling pathways regulate many integrated physiological functions of the nervous system including neuronal development, synapse formation, synaptic plasticity, memory storage, and cognition [45,46]. Accordingly, abnormalities in mTOR activity are linked with severe deficits in nervous-system development [47,48]. Cognitive dysfunction is the most typical outcome of abnormal neural development in premature infants [49,50]. A study on premature rats suggests that this effect may be due to suppressed mTOR signaling in the hippocampus [51]. Experimental upregulation of mTOR signaling promotes retinal ganglion cell survival and axon regeneration after optic nerve crush injury [52,53]. Zhang and coauthors [54] demonstrated a critical role of muscle mTOR catalytic activity in the regulation of whole-body growth and homeostasis. They proved that treatment of skeletal muscle with mTOR catalytic inhibitors may have detrimental effects. Based on the results of a study on photoreceptor cells, Fan et al. [55] suggested that inhibiting mTOR signaling might cause cells to enter lower and more stable bioenergetic states, in which neurons have greater resistance to various insults. We hypothesized that a steady decline of mTOR activity in the retina of OXYS rats may be implicated in the alterations of the balance of neurotrophic factors in the retina [56] and in the mitochondrial dysfunction typical for OXYS rats [27,57]. As we previously reported, therapeutic effects of long-term SkQ1 consumption on the AD-like pathology in OXYS rats are associated with improvement of the mitochondrial apparatus and better neurotrophic supply [9,27].

mTOR performs important function in both innate and adaptive immune responses [58]. It can be speculated that a steady decline of mTOR activity in the OXYS rats’ retina is involved in the imbalance of the retinal immune responses that is seen specifically in OXYS rats [20,59], during accelerated thymus involution and a decrease in the activity of the T-cell components of the immune system, both of which may be slowed down by SkQ1 [60].

We previously observed a therapeutic action of SkQ1 on the ultrastructural pathological alterations in the RPE of OXYS rats decreasing of lipofuscin content [61]. Furthermore, SkQ1 could compensate autophagy disturbance by chaperone-mediated proteolysis. Recently, we found that the treatment with SkQ1 from 1.5 to 4 months of age (250 nmol/kg) increases the level of β-crystallin (a small heat shock chaperone) in the retina of OXYS rats [16]. The effect of SkQ1 was reflected in the attenuation of neurodegenerative changes in photoreceptors and better circulation in choroid blood vessels in the retina of OXYS rats [16]. Treatment with SkQ1 suppressed disease progression, even if SkQ1 administration was started after some rats had already developed stage 1 retinopathy.

In this study, we confirmed the previously identified ability of dietary supplementation with the mitochondria-targeted antioxidant SkQ1 (starting at the preclinical stage of AMD-like pathology) to suppress the development of the clinical signs of AMD in old OXYS rats. We suggest that the effect of SkQ1 on Aβ is a potential molecular mechanism behind the normalization of the retinal structure and function. Perhaps another crucial action may be SkQ1-mediated suppression of the mTOR pathway activity in the retina. This action may participate in the beneficial effects against AMD-like pathology in OXYS rats. In the retina of OXYS rats, we detected reduced mTOR activity, whose contribution to the development of AMD-like pathology should be investigated further. Nevertheless, it is obvious that inhibition of mTOR signaling activity, which plays a key part in aging and age-related diseases, can be regarded as a new mechanism of the prophylactic effect of SkQ1. The results of this study confirmed that dietary supplementation with the mitochondria-targeted antioxidant SkQ1 could be a good prevention strategy to maintain eye health and possibly a treatment of AMD.

## 5. Limitations of the Study

A limitation of OXYS rats and of other rodent models of AMD is that they do not have a macula, nor do they have an area of high cone density analogous to the fovea [62]. In addition, at the base of the RPE, rodents do not develop deposits that have composition similar to that of drusen in humans, perhaps because of a difference between rodents and humans in the mode of transport of lipids across the RPE [62]. In contrast to the drusen that develop in humans, according to many studies, the drusen-like deposits in rats are not located on the basal RPE [62]. Furthermore, OXYS rats are albinos. Therefore, the fundus pattern is different from that typical for AMD as we demonstrated here and earlier [15,34,63]. Nevertheless, OXYS rats spontaneously develop the major signs of AMD and fairly well reproduce the stages of the disease: dystrophic alterations of the RPE, thinning of the neuroretina, and impairment of choroidal microcirculation [17,20,21]. It should be noted that none of the available animal models reproduces all the features of human AMD, and therefore cannot be considered a representative model of AMD as a complete disease [64]. Moreover, in humans, there are many AMD phenotypes as a result of the genetic background, different lifestyles, and environmental risk factors.
